# A Comparison Study between Traditional and Deep-Reinforcement-Learning-Based Algorithms for Indoor Autonomous Navigation in Dynamic Scenarios

**DOI:** 10.3390/s23249672

**Published:** 2023-12-07

**Authors:** Diego Arce, Jans Solano, Cesar Beltrán

**Affiliations:** Engineering Department, Pontificia Universidad Católica del Perú, San Miguel, Lima 15088, Peru; jans.solano@pucp.edu.pe (J.S.); cbeltran@pucp.edu.pe (C.B.)

**Keywords:** comparison study, indoor autonomous navigation, mobile robots, dynamic scenarios, traditional navigation, DRL-based navigation

## Abstract

At the beginning of a project or research that involves the issue of autonomous navigation of mobile robots, a decision must be made about working with traditional control algorithms or algorithms based on artificial intelligence. This decision is not usually easy, as the computational capacity of the robot, the availability of information through its sensory systems and the characteristics of the environment must be taken into consideration. For this reason, this work focuses on a review of different autonomous-navigation algorithms applied to mobile robots, from which the most suitable ones have been identified for the cases in which the robot must navigate in dynamic environments. Based on the identified algorithms, a comparison of these traditional and DRL-based algorithms was made, using a robotic platform to evaluate their performance, identify their advantages and disadvantages and provide a recommendation for their use, according to the development requirements of the robot. The algorithms selected were DWA, TEB, CADRL and SAC, and the results show that—according to the application and the robot’s characteristics—it is recommended to use each of them, based on different conditions.

## 1. Introduction

Intelligent systems are an area of computer science that seeks to solve complex and multidisciplinary problems in an automatic way by capturing information from which it can perform actions and evaluate its result, in order to learn from its experience and improve its performance and efficiency. These systems can be applied in robotics for autonomous-navigation purposes, where the use of conventional automatic control techniques is very complex. The sub-areas with the most potential are Reinforcement Learning (RL) and Deep Learning (DL), through the combination of which it is possible to solve highly complex problems from the training of computational frameworks based on rewards for taking a correct action or penalties for incorrect actions.

The navigation function within the robotics area is one of the most important. It allows the system to move through an environment safely, in order to guarantee its arrival at the destination, taking into account environmental restrictions. In general, the navigation function of a mobile robot is achieved through Point-to-Point-(P2P) movement and obstacle avoidance [1].

The navigation function within a robot is carried out from a traditional approach mainly by executing two tasks: Simultaneous Localization and Mapping (known as SLAM). These tasks make it possible to generate a virtual map of the environment through which the robot is going to move. Then, using the information from this map, it is possible to determine the position of the robot and to plan the path that it must follow [2].

From a general perspective, it may not be very complex to implement a navigation function in the robot. Nevertheless, each aspect that this function involves turns out to be challenging, and a research topic may be necessary for each of the aspects when using the traditional method.

The implementation of the navigation function using the traditional method usually causes large computational errors or requires equipment with high computational capacity. In the absence of the correct equipment, the calculation errors accumulate during the execution of the mapping, positioning and planning tasks, in time causing inaccurate movement. As previously described, this traditional method is mainly based on information from the virtual map, which is usually very sensitive to noise. This turns out to be very limiting, especially when trying to carry out applications in unknown or dynamic environments [1].

The integration of Deep Reinforcement Learning (DRL) algorithms in the autonomous-navigation functions of the robot allows the determination of the optimal policy capable of conducting the movement of the robot from an initial position to a final position through continuous interaction with a static or dynamic environment. Different DRL algorithms—such as DQN, DDPG, PPO and their variants—have been applied to performing robot navigation [1].

One of the advantages of using DRL for navigation is that it does not require maps or precision sensors. On the other hand, as RL is based on trial-and-error principles, it is necessary to initially carry out virtual training, because during this stage collisions will inevitably be generated between the robot and obstacles. This process is carried out in simulation environments with a high level of similarity to real environments and allows for defining the optimal DRL algorithms for implementation in a real robot.

Autonomous navigation of ground-mobile robots is a highly studied topic, due to its potential application in multiple areas, such as logistics, mining, medicine, care and other fields [3]. In recent years, these studies have been focused on the use of Deep Reinforcement Learning (DRL) to provide autonomous-navigation capacity to robots being validated with tests in simulated environments and in real environments.

Studies where DRL is used for robot navigation have been carried out both indoors [4,5,6,7] and outdoors [8,9], where the algorithms for the development of computational frameworks require different considerations. Furthermore, the research carried out shows that it is possible to consider providing autonomous-navigation capacity to a robot by means of DRL from the use of information from known maps [10,11], partial information from maps [12] or without information on the environment [13,14,15,16,17,18], for which different algorithms can be used. Another important aspect to take into account is the need to interact in environments with non-stationary elements. Therefore, some studies will be taken as a reference in which the interactions of the robot with people [8,9] and other non-stationary elements are taken into account [11,19] during robot navigation.

Several published studies have focused on the development of new navigation techniques and navigation algorithms, and a few that compare traditional and DRL-based algorithms. Despite this, there is no clear way to determine whether it is better to implement a traditional navigation or a DRL-based navigation approach, especially for ground-mobile robots in dynamic scenarios. This article proposes a comparative study between both autonomous-navigation approaches, by identifying the most effective algorithms and applying them to virtual scenarios with a real robot, in order to measure specifically determined parameters. Based on the results obtained, it will be possible to suggest in which cases it is recommended to use a traditional approach and in which cases a DRL-based approach.

This research started with a review of the different autonomous-navigation algorithms applied to mobile robots. Based on this, the most suitable ones were identified for the cases in which the robot must navigate in dynamic environments. Subsequently, a comparison of these algorithms (traditional and DRL-based) was performed, using a robotic platform in a virtual environment, to evaluate their performance and identify their advantages and disadvantages. At the end of the article, an analysis of the compared algorithms is presented and linked to practical applications. This paper aims to contribute in the field of autonomous navigation by providing a recommendation for the development of new autonomous robots and the selection of an autonomous-navigation algorithm for dynamic environments.

This article is structured into five sections. Section 1 introduces the reader to the general topic of the article. Section 2 presents a brief description of the background studies and relevant information related to autonomous navigation, and a review of traditional navigation and DRL-based algorithms. Section 3 describes the methodology applied for the comparative study, detailing the robot characteristics, the simulation environment, the algorithms applied and the parameters measured. Section 4 presents and discusses the results obtained from the comparative study. Section 5 presents the conclusions of this study and outlines future work based on this research.

## 2. Background Studies

**Figure 1:**
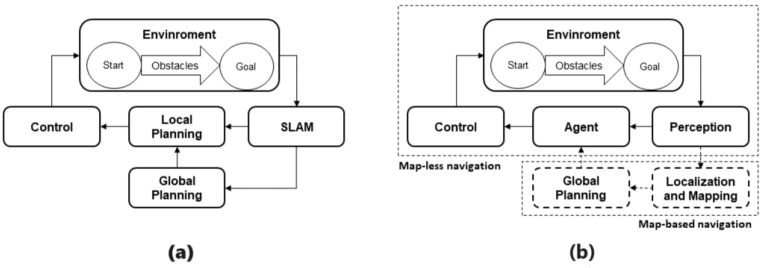
Representation of autonomous navigation: (**a**) Traditional algorithms. (**b**) DRL-based algorithms.

This section presents the information relevant to understanding the autonomous-navigation process for a ground-mobile robot. Two approaches are taken into consideration: traditional navigation algorithms and DRL-based navigation algorithms. The application of DRL for robot navigation modifies the traditional approach, causing the new approach to be determined as the one presented in Figure 1. This diagram presents the navigation based on DRL and shows the interaction between the agent and the environment, where the agent replaces the localization process, map generation and local planning. When working with environments with simple structural obstacles (simple environments) this composition is effective and does not require more complexity, but if working with complex structural obstacles (complex environments) it is necessary to include other additional processes of traditional navigation. For these cases, an analysis of the environment is used to determine global planning that allows for having intermediate reference points that will be used by the agent to move the robot [20].

### 2.1. Simultaneous Localization and Mapping (SLAM)

In recent years, significant advances have been made in visual SLAM techniques. However, they still face challenges in achieving reliable localization, especially in textureless environments. On the other hand, LiDAR-based SLAM provides more robust localization, because 3D information is directly captured from a point cloud [21]. Therefore, the following subsection will explain the main algorithms based on the use of laser sensors that solve the Simultaneous-Localization-and-Mapping task.

#### 2.1.1. Gmapping

Gmapping improves the Rao–Blackwellized Particle Filter (RBPF), where each particle represents a possible position of the robot, allowing for the construction of the map and random distribution in the environment [22]. These particles propagate based on the robot’s movement and, subsequently, update the map and particle weights according to the similarity between their distribution and sensor measurements. One advantage of Gmapping over the RBPF algorithm is the reduction in computational cost achieved by decreasing the number of particles in each iteration [23].

To accomplish this, Gmapping employs adaptive sampling, using Equation (1), where *N* represents the number of particles, $w^{i}$ denotes the weight associated with particle *i* and $N_{\mathrm{threshold}}$ is the threshold used for sampling:

(1) $$N_{\mathrm{threshold}}=\frac{1}{\sum_{i=1}^{N}{\left(w^{i}\right)}^{2}}$$

The value of $N_{\mathrm{threshold}}$ measures the dispersion of the particle set. In other words, a larger value indicates a more concentrated particle set and lower estimation error [21]. Therefore, when $N_{\mathrm{threshold}}<\frac{N}{2}$, the sampling is performed, and particles with low weight are replaced by samples with higher weight.

#### 2.1.2. HectorSLAM

This technique allows for Simultaneous Localization and Mapping, not only in ground vehicles but also in aerial ones. Additionally, it presents low computational cost, making it suitable for low-resource processors. The algorithm consists of two main subsystems, one related to the 3D navigation system and the other focused on the 2D SLAM. The first subsystem uses inertial information from an IMU to provide an orientation estimation to the 2D-SLAM subsystem [24]. It is worth noting that, unlike other SLAM techniques, it requires synchronization between both subsystems through a real-time critical controller [21].

The 2D-SLAM subsystem uses scan matching as its fundamental component, which is a process for finding correspondences between the sensor-scanned information and the environment information provided by the map. For this purpose, it employs Equation (2), through which it finds the orientation and translation of the laser beam’s endpoints, allowing alignment of one measurement with another and with the map created up to that point [24]:

(2) $$\varepsilon^{*}=\arg\min_{\varepsilon }\sum_{i=1}^{n}{\left[1-M\left(\varepsilon \right)\right]}^{2},$$

where ε represents the transformation, i.e., the orientation and translation, while $\varepsilon^{*}$ corresponds to the transformation that generates the best alignment of the laser scan with the map and, therefore, minimizes the sum of the scan error in the formula [21]. On the other hand, M(ε) is the transformation from a global coordinate system to one relative to the map.

The 3D-navigation subsystem consists of sensor fusion, which, through an extended Kalman filter, can estimate the 3D position and orientation of the robot. This information is projected onto a 2D plane and enters the SLAM subsystem for scan matching. Furthermore, in the opposite direction, the information estimated by SLAM improves the estimation of the navigation subsystem [24].

#### 2.1.3. Karto-SLAM

Karto-SLAM is a Simultaneous-Localization-and-Mapping algorithm based on graph optimization, in which it calculates all the states *x* taking into account the time range [21]. Thus, for the calculation of the latter, the control signals u and the previous *x* states are taken into consideration. In that sense, the main difference with respect to the particle filter is that the latter estimates only the state that is optimal at the current time $x_{t}$, while the former estimates a global configuration.

In the context of Karto-SLAM, the graph optimization uses as nodes the robot positions and is connected by constraints that are obtained from sensor observations [25]. In that sense, the resulting graph is large and, therefore, the computational cost increases. In view of this, Karto-SLAM employs a method called Sparse Pose Adjustment (SPA), which allows fast convergence even in complex environments. Moreover, when a scan-matching system is added to the SPA, a real-time mapping system can be obtained, as the graph-based approach allows for the addition and removal of details to the map without any problem [25].

### 2.2. Global Planning

Currently, optimal wayfinding is a widely studied topic with varied applications. For example, application or Web maps provide optimal routes to destinations that are entered by a user. To do this, this service first requires converting the map to a graph. From this, algorithms are used to find optimal solutions to planning problems in given environments. These algorithms take into account direct information from the environment through sensors and consider different aspects for optimization, such as execution time, route distance and obstacle avoidance, among others. In this sense, the main algorithms used to solve this task will be detailed below.

#### 2.2.1. Artificial Potential Fields

The potential-fields method has as its main advantage its speed and effectiveness in solving the global-planning problem. This method suggests considering an initial approximation of the route to follow, in order to avoid stagnation at a local minimum, and then correcting it using the potential fields until an optimal solution is found [26]. The obstacles correspond to repulsive fields and the goal to attractive ones, so that the environment results from the vector sum of these fields.

Using this method, the robot must move in the direction in which the potential field decreases, i.e., in the direction of the field gradient evaluated at the robot’s position. The above is formulated by Equation (3), where an additional force F is introduced that drives the robot to follow the direction in which the potential field decreases [27]:

(3) $$\vec{F}\left(q\right)=-\nabla U\left(q\right)=-\left(\nabla U_{\mathrm{atr}}\left(q\right)+\nabla U_{\mathrm{rep}}\left(q\right)\right),$$

where *U* represents the resulting potential field and q the current position of the robot. From the above, path planning is achieved by defining a unit vector pointing to the gradient and a parameter δ defining the robot’s feed size [27], as illustrated in Equation (4):

(4) $$q_{i+1}=q_{i}+\delta_{i}\frac{\vec{F}\left(q\right)}{∥\vec{F}\left(q\right)∥}.$$

#### 2.2.2. A Star (A*)

This technique is based on the Dijkstra algorithm, which is detailed in the pseudocode Algorithm 1. First of all, an infinite distance is assigned to all nodes except the origin node, which is assigned a distance of 0. Then, the unvisited node with the shortest distance is selected and marked as visited. Furthermore, the distances of adjacent nodes are updated by summing the distance of the selected node and the weight of the connecting edge, only if this accumulated distance is less than the currently stored distance for the adjacent node. These selection and update steps are repeated until all nodes have been visited or until there are no nodes with finite distances left. Finally, the shortest path from the origin node to any other node is obtained by following the updated distances.

On the other hand, A* differs from Dijkstra’s algorithm in the node-estimation function, as the latter finds the optimal route considering only the cumulative cost, whereas A* takes into account a heuristic estimation that allows for solving the problem more efficiently. For the cost estimation using the heuristic function, first the cost $c_{\left(\mathrm{start},i\right)}$ between the initial node and a node *i* is found; subsequently, the nearest neighbor *j* is searched and the distance $d_{\left(i,j\right)}$ between them is estimated; and, finally, the distance $d_{\left(j,\mathrm{goal}\right)}$ between node *j* and the node corresponding to the goal is estimated [28]. The total cost takes into account these three parameters, as can be seen in Equation (5):

(5) $$C_{i}=c_{\mathrm{start},i}+\min_{j}\left(d_{i,j}+d_{j,\mathrm{goal}}\right).$$

**Algorithm 1:** Dijkstra’s algorithm.
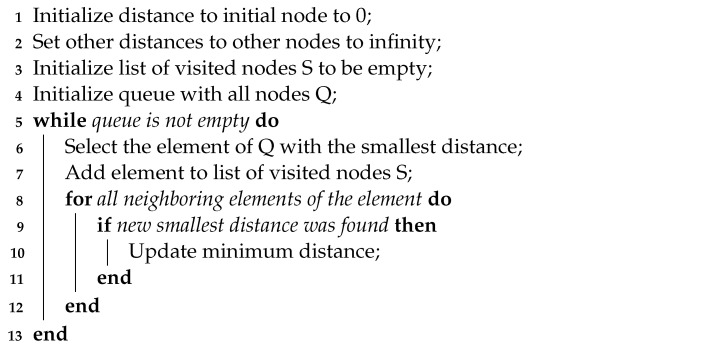


### 2.3. Local Planning

This section describes three local planners commonly used in the literature for robot navigation: DWA, TEB and MPC. Moreover, a comparison of their performance is presented.

#### 2.3.1. Dynamic-Window Approach (DWA)

This algorithm is an approach used in autonomous navigation, based on the velocity space of the robot and its optimization, to obtain an optimal local route. Unlike other methods, it takes into consideration the dynamics of the robot, which, within its approach, considers the physical limitations that the robot may have, in terms of its velocity and acceleration [29].

**Figure 2:**
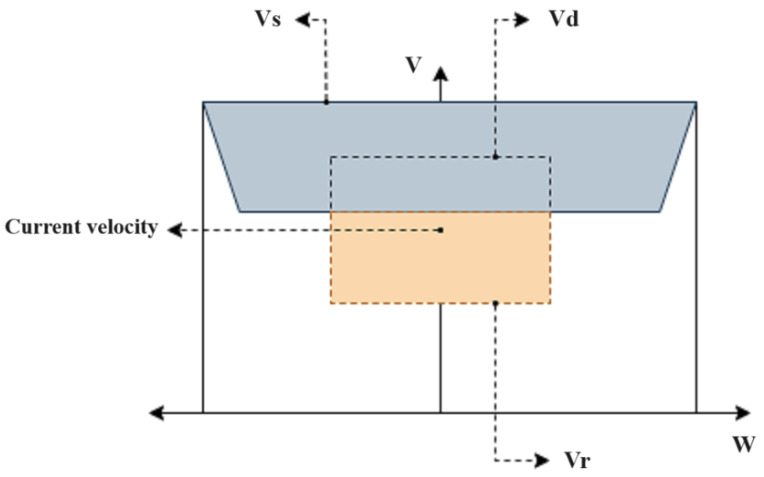
Dynamic Window.

The DWA method starts from a velocity search for the robot in a two-dimensional space, as it considers only curvatures that are generated by pairs of linear and angular velocities (v,w). Figure 2 shows the velocity space $V_{s}$ and the dynamic window $V_{d}$ of a robot, where the gray area corresponds to the set of velocities that the robot cannot reach without stopping before a collision with an obstacle. The rest corresponds to admissible velocities, while the yellow zone, which is included within $V_{d}$, corresponds to $V_{r}$, the velocities that can be reached considering the physical limitations of the robot’s velocity and acceleration.

After generating a feasible velocity space window for the robot, a cost function is generated to choose the velocity that the robot should follow, using Equation (6), shown below [30]:

(6) G(v,w)=σ(α·direc(v,w)+β·dist(v,w)+ϕ·vel(v,w)).

Of all the trajectories generated by $V_{r}$, the first component of the formula evaluates the alignment of the robot with the direction of the goal. After sampling the velocity space $V_{r}$, the curved trajectory of the robot when taking that velocity is simulated, to measure the angle towards the goal of this new estimated position. Then, the formula considers the distance to the nearest obstacle and, finally, the current velocity of the robot. From this cost function, the velocity that minimizes it and, therefore, optimizes the local planning is chosen.

#### 2.3.2. Time Elastic Band (TEB)

The TEB algorithm arises from the possibility of a robot finding itself in an incomplete environment or with dynamic obstacles, where global gliders do not provide an adequate solution. In that sense, this approach takes into account the path generated by a global glider and is based on the idea of the elastic band, in which the initial path is deformed, considering it as an elastic band subjected to external forces that swings while keeping a certain distance from obstacles [31].

Unlike the elastic band, the TEB not only takes into account the intermediate states, such as orientation $\beta_{i}$ and the position of the robot $x_{i}$, but adds the time intervals between states $\Delta T_{i}$. Within the objective functions of the TEB are conditions related to the dynamics of the robot and others regarding the efficiency or speed in fulfilling the trajectory [31]. In the following paragraphs, these aspects will be detailed.

First, the TEB seeks that the partial points of the trajectory generate an attractive force towards the rubber band, while the obstacles repel it [31]. This is achieved by obtaining the minimum distance to a waypoint or obstacle during the trajectory. Subsequently, objective functions that restrict the values of these distances according to the type of mark are considered, so that the distances to the obstacles are as large as possible and the tracking error of the route is minimal.

Likewise, the TEB algorithm obtains the shortest route by generating internal forces that deform the elastic band [31]. In the case of the TEB, in addition to meeting that constraint, it is desired to obtain the fastest route, which is achieved by minimizing the summation of times between states $\Delta T_{i}$. Finally, the dynamic constraints of velocity and acceleration are achieved in a similar way for the case of intermediate points and obstacles, as the minimum and maximum constraints of these are taken into account.

#### 2.3.3. Model Predictive Control (MPC)

Model Predictive Control (MPC) is a control method that operates in discrete time. It employs a model of the system, optimizes a problem according to a given cost function and then outputs control signals [29]. In the particular case of the local planner, MPC is used to plan and control the movement of the robot in a local environment. It is also responsible for generating smooth and safe trajectories that allow the robot to navigate efficiently and to avoid obstacles.

(7) $$\left[\begin{array}{c} x_{k+1|k}\\ x_{k+2|k}\\ \vdots \\ x_{k+H|k} \end{array}\right]=\left[\begin{array}{c} A\\ A^{2}\\ \vdots \\ A^{H} \end{array}\right]x_{k}+\left[\begin{array}{cccc} B & 0 & \cdots & 0\\ AB & B & \cdots & 0\\ \vdots & \vdots & \ddots & \vdots \\ A^{H-1}B & A^{H-2}B & \cdots & B \end{array}\right]\left[\begin{array}{c} u_{k|k}\\ u_{(k+1)|k}\\ \vdots \\ u_{(k+H-1)|k} \end{array}\right]$$

MPC uses the mathematical model shown in Equation (7), which describes the dynamics of the robot and the ability to predict future states of the system in discrete time [29], where *H* represents the time period of the model prediction and *u* represents the control variable. It should be noted that the model shown is linear; however, it can be applied to nonlinear systems, previously performing a linearization around the state of the robot. From these predictions, MPC seeks to find a sequence of control actions that optimize a defined cost function, which may be restricted to the physical limitations of the system, such as the speed and acceleration of the robot. The cost function may present different criteria, such as minimizing the distance traveled, arrival time or maximizing obstacle-avoidance capability.

#### 2.3.4. Review of Local Planners

Table 1 shows a comparison of the revised State-of-the-Art of local-planning algorithms. The research found in this regard is mainly focused on updates of these algorithms. It should be noted that most of these new enhancements are focused on improved performance in dynamic environments.

### 2.4. Reinforcement-Learning Review

Global-planning methods represents a problem when the map of the environment is not available or is inaccurate, which is precisely the situation of most autonomous-navigation problems [30]. In view of this, local planners solve this problem, as they are able to adapt quickly to real-time variations of the environment. Also, because their focus is limited to a portion of the environment, they require less computational resources, which allows them to update the world at an appropriate frequency, as measured by sensors.

#### 2.4.1. Deep Reinforcement Learning

**Figure 3:**
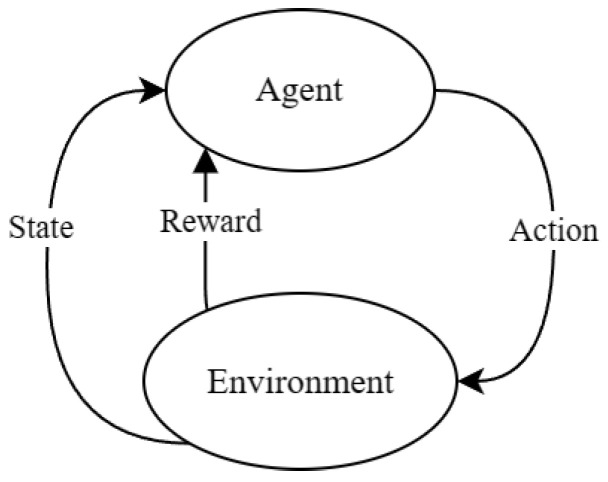
Reinforcement-Learning Scheme.

Reinforcement Learning (RL) is a branch of machine learning, in which an agent learns a certain task through trial and error. An RL system has three main components: policy, reward and value functions. The policy function allows mapping the states in a given environment to the actions to be taken. The reward function corresponds to the signal that evaluates the quality of those actions. The value function estimates the total cumulative reward expected for a given state [38]. In Figure 3, the above is illustrated, where an agent performs an action in a given environment, receives a reward and a new state. It should be noted that the agent’s main objective is to maximize the accumulated reward through interaction with the environment.

On the other hand, Deep Learning (DL) emerges as a tool that, through the use of multilayer approximations of nonlinear functions [39], allows for solving different tasks of the machine-learning branch. In that sense, Deep Reinforcement Learning (DRL) is the intersection of the DL and RL fields.

#### 2.4.2. Review of Deep-Reinforcement-Learning Algorithm Classification

**Figure 4:**
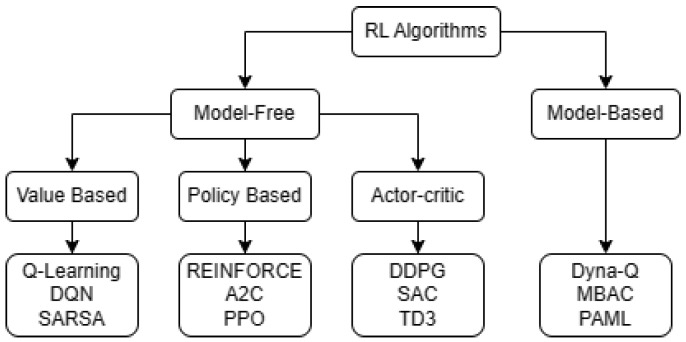
Taxonomy of RL algorithms.

DRL algorithms can be classified into two main approaches: model-based and model-free (as shown in Figure 4). The difference between the two lies in the fact that model-based algorithms elaborate a model of the environment that allows the agent to predict how the environment will behave in response to its actions, whereas model-free agents learn from the interaction with the environment, without knowing its behavior. It should be noted that the following chapters will focus on the model-free algorithms, because, otherwise, the modeling of an uncontrolled environment is a problem.

Within the model-free approach are the algorithms based on the policy, value function and actor–critic. The value function is estimated to then derive the policy. In that sense, giving the function a state *s* and an action *a*, by means of the following formula the optimal policy is obtained [40]. That is, the action that maximizes the value function, on the basis of a given state, is taken as shown in Equation (8):

(8) $$a=\arg\max_{a}Q\left(s,a\right),$$

while the policy-based algorithms directly optimize it without estimating the value function initially. For this, it is a matter of finding a series of parameters θ, which optimizes π(s,a|θ); this, being a more direct method, allows greater stability and reliability in the results [40]. Finally, the actor–critic algorithms combine the methods by value function and policy and obtain a neural network of the actor type that refers to the policy while the critic corresponds to the value function of the current policy Q(s,a;θ) [41]. The critic evaluates the actor’s performance and provides feedback. Meanwhile, the actor uses the feedback to improve the training of the policy.

### 2.5. Review of DRL-Based Navigation Algorithms

Table 2 shows a comparison of autonomous-navigation algorithms for mobile robots employing DRL techniques reviewed in the literature. Here, it is worth highlighting that the application scenarios are mainly divided into three: static, dynamic and social navigation. The latter is focused on navigation in highly dynamic environments, such as a place with many passers-by. According to the State-of-the-Art for social navigation, there is an additional perception to traditional sensors, which is based on a static- or dynamic-agent level. On the other hand, the action space can be varied depending on the dynamic constraints of the robot or algorithm to be used. Finally, it is emphasized that for robust social navigation, prior information about the environment, such as a map, is generally required.

## 3. Methodology

This section describes the methodology followed in order to execute this research. First, the simulation configuration describes in detail the robot characteristics and the simulation environment. Then, the ROS-navigation stack is detailed, focusing on the mapping, localization and path-planning steps. Finally, the implementation of the local-planners algorithms and the mapless-DRL-based algorithms are explained.

### 3.1. Simulation Configuration

In the past, the implementation of robots required to be evaluated once they were fully implemented. This entailed excessive development costs, in case of unforeseen events or failures. Currently, there are different 3D-simulation environments capable of faithfully representing physical and dynamic aspects of the real world. There are also tools that allow the speeding up of the tasks that are most demanded in the field of robotics. Therefore, these two aspects related to the implementation of this project will be detailed below.

#### 3.1.1. Robot Description

**Figure 5:**
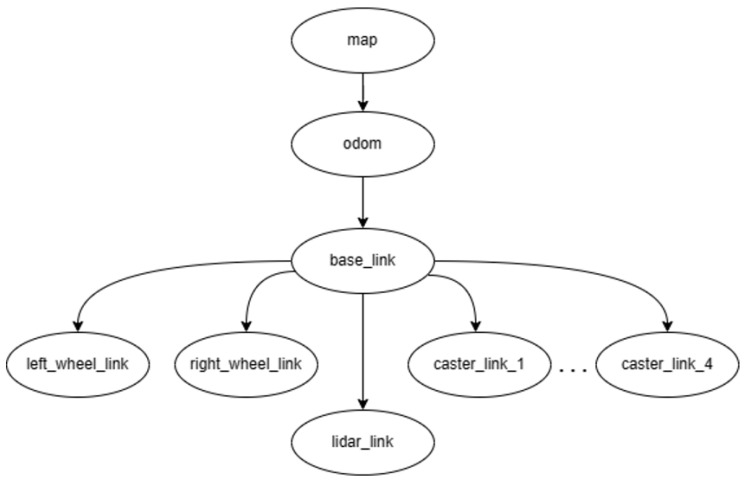
Robot URDF tree diagram.

**Figure 6:**
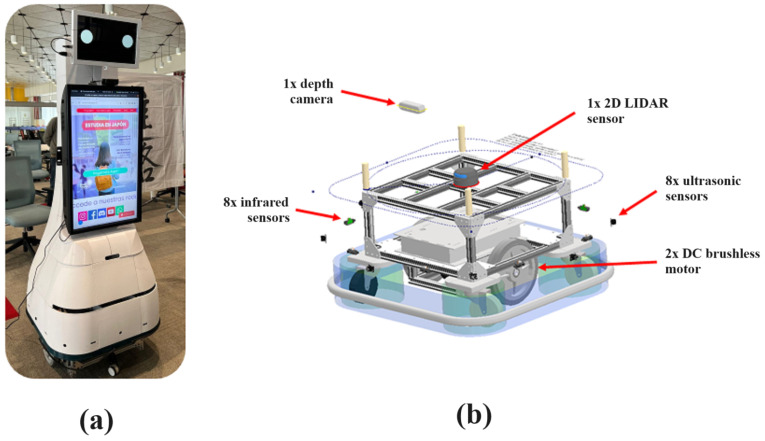
Robot used for the study: (**a**) Real robot image. (**b**) Internal components of robotic platform.

The description of the robot in the simulation was based on the tree diagram in Figure 5, where the connections between the main components of the robot and the transformations generated when creating the robot model using the Unified Robot Description Format (URDF) are defined. It can be highlighted that the robot used a differential drive, in which there were four pivoting wheels (as shown in the detailed design of the robotic platform in Figure 6). Likewise, the laser implemented in the simulation had a scanning range of 240°. On the other hand, it should be noted that the odometry of the real robot is calculated by means of a sensor fusion of an inertial sensor and encoders on the wheels. However, for simulation purposes, the simulator was in charge of the respective calculations.

#### 3.1.2. Simulation Environment

**Figure 7:**
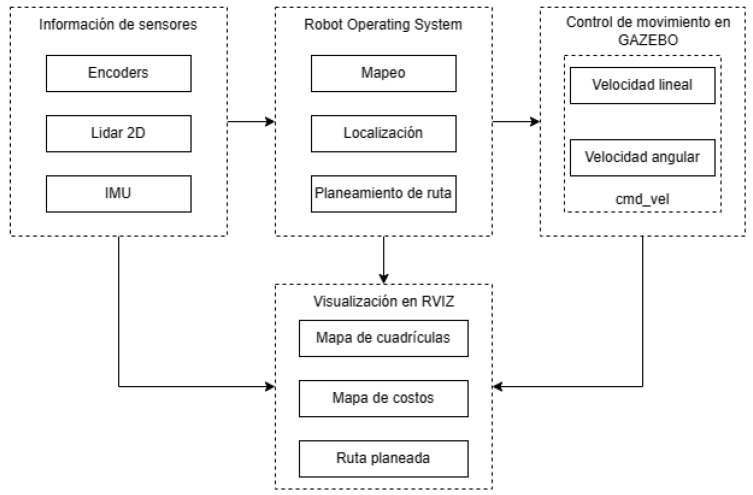
ROS-navigation stack for robot simulation.

For the implementation of classical-navigation algorithms, the Robot Operating System (ROS) framework and the Gazebo simulator were used. The latter provided information from the environment that was measured by the sensors and used by the ROS-navigation package, which consisted of mapping, localization and route planning, both global and local. As shown in Figure 7, this package sent speed commands to the robot that was simulated in Gazebo. Also, the information on grid maps, costs and the planned route were visualized in RVIZ.

### 3.2. ROS Navigation Stack

The ROS-navigation package facilitates the work of implementing each of the algorithms necessary for the autonomous navigation of mobile robots of a large number of configurations. Figure 7 illustrates the main components of the package and how they interact with each other. It highlights that there are specific nodes of the robotic platform, such as sensors or odometry source, which are detailed above. On the other hand, there are the route planning, mapping and localization nodes, which are detailed below.

#### 3.2.1. Mapping

In order to obtain a map for navigation, a 3D environment was designed, in which the different navigation algorithms were evaluated. This environment was built in Gazebo 9.0 and was intended to simulate a dynamic social environment such as, for example, a shopping mall. Therefore, it had corridors, entrances and rooms, as well as maximum dimensions of 27.5 × 34 m.

**Figure 8:**
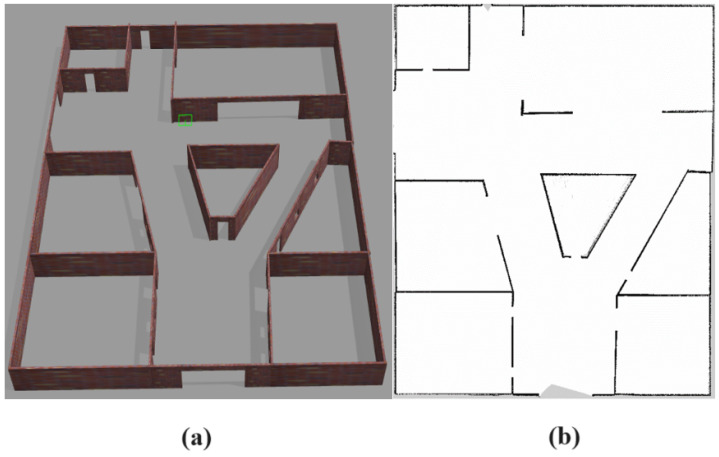
Mapping of the environment: (**a**) 3D environment. (**b**) Occupancy grid map.

Figure 8a illustrates the recreated 3D environment, while Figure 8b shows the occupancy map. For the latter, the Gmapping package was used; it should be noted that only a 2D laser sensor was used for sensory perception.

#### 3.2.2. Localization

**Figure 9:**
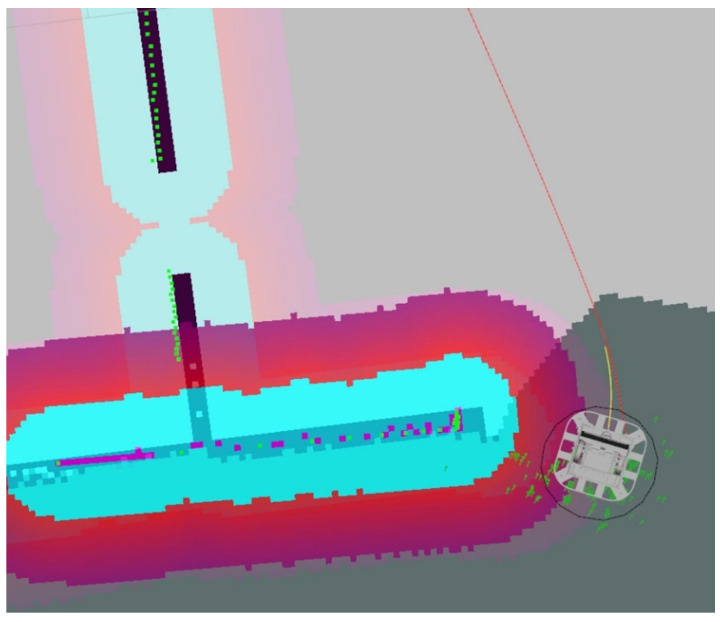
Robot localization algorithm during simulation.

The AMCL-ROS package was employed for the robot localization, i.e., for the robot position and orientation estimation. For this purpose, the Adaptive-Monte-Carlo-Localization-(AMCL) algorithm was employed, which subscribed to the 2D-laser-sensor topic, robot transformations and map. It is worth noting that the package improved its localization accuracy as more time elapsed. The algorithm was based on a particle filter, to represent the hypotheses of the most probable positions of the robot. When the robot performed a measurement, the similarity of that information to the information provided by the map was evaluated and, in this way, after the robot’s movement, the new state of the particles was predicted. Thus, the particle distribution was adaptively adjusted to the information from the environment and sensor measurements. Figure 9 shows the localization package in the operation of the robot, where the green arrows correspond to the particles generated from the filter.

#### 3.2.3. Path Planning

The global route planning was generated by the Navfn algorithm, which used Dijkstra’s algorithm to find the most optimal route. It should be noted that there was the possibility of it being modified to use the A* algorithm, but the results using Dijkstra were similar and did not affect navigation, because the local planner was responsible for generating the avoidance of obstacles not considered in the map. Finally, Figure 9 illustrates the route generated by the global and local planners, as a black line and a yellow line, respectively.

It is worth mentioning that achieving optimal navigation performance involved fine-tuning parameters related to the inflation layer of the cost map. As illustrated in Figure 9, the cost associated with cells was heightened, as indicated by the expanded size of the sky-blue contour encircling the map. This adjustment resulted in the generation of a more secure route for the robot, steering it away from potential obstacles and enhancing overall safety.

### 3.3. Local-Planners Algorithms Implementation

Table 3 shows the main modified parameters of the local planners. Initially, we can highlight the values that correspond to the physical limits of the robot, such as velocity and acceleration. On the other hand, the tolerance of distance and angle to the goal were 0.1 m and 0.2 rad, respectively.

#### 3.3.1. Dynamic-Window Approach (DWA)

The first important parameter of the DWA algorithm is the sim_time, as it corresponds to the time that the glider will have to obtain an optimal solution. In particular, a value of 2.5 s was chosen, as it was an adequate time in which to solve situations that might require a high processing time and, at the same time, did not reach high times. On the other hand, as for the vx_samples and vth_samples parameters, values of 20 and 40, respectively, were taken. This was due to the fact that the higher the number of samples, the higher the computational cost and that, in general, the rotation task was more complex than the translation task in the x-axis. Finally, other relevant parameters corresponded to the path-cost function implemented in ROS, shown in Equation (9):

(9) $$\begin{array}{cc} \cos t & =\text{path\_distance\_bias · distance\_to\_path}+\\ \; & \;\text{goal\_distance\_bias}\cdot \text{distance\_to\_local\_goal}+\\ \; & \;\text{occdist\_scale}\cdot \text{maximum\_obstacle\_cost\_on\_path}, \end{array}$$

where the parameter path_distance_bias corresponded to how close the path was to the global route, goal_distance_bias corresponded to the weight with which the robot would approach the local goal instead of the global route and occdist_scale was the weight with which the robot avoided obstacles [46]. These parameters were set to those shown in Table 1, because of their positive results for the simulated robotic platform.

#### 3.3.2. Time Elastic Band (TEB)

**Figure 10:**
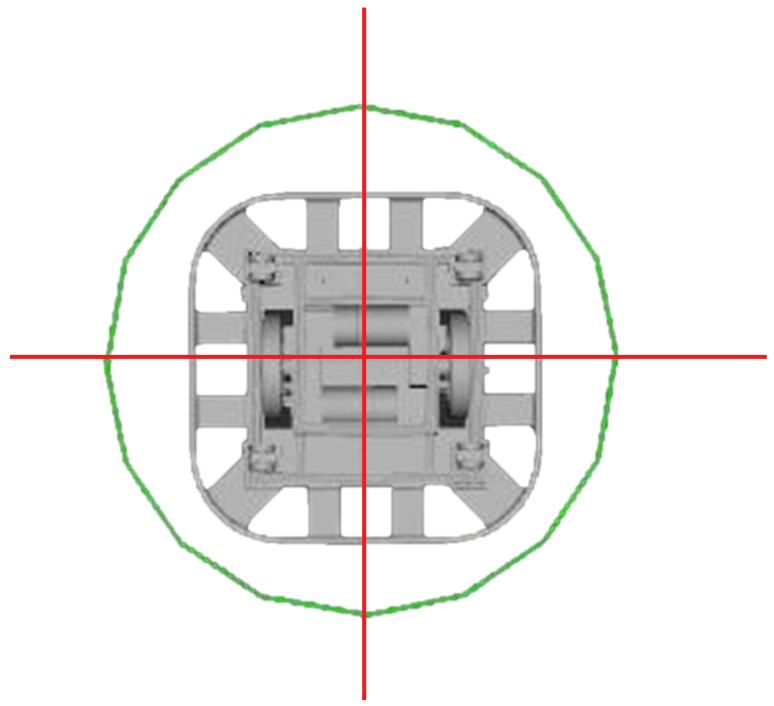
Robot base footprint.

Unlike the DWA, the TEB algorithm allows reverse motion, so one of the differentiating parameters with respect to the DWA in the dynamics of the robot was max_velocity_x_backwards, and it was configured to have the same linear velocity as the frontal motion. Also, the 2D footprint model for the robot was redefined for this local glider, because unlike the footprint used for the cost map it was intended to be a simpler representation than the 2D projection of the CAD model of the robot. Figure 10 illustrates the comparison of the footprints for both situations.

The main reason behind this was that the cost map footprint was used to detect a possible collision and, therefore, required precision in its implementation, while TEB was used for optimization purposes; the complexity of the original figure was reduced to a circular one, to reduce computational time. With respect to obstacle avoidance, parameter values were defined by trial and error until adequate results were obtained in confined, wide or obstacle spaces. In this sense, the main modified parameters were min_obstacle_dist and include_dynamic_obstacles, with values of 0.4 m and True, respectively.

### 3.4. Map-Based-Deep-Reinforcement-Learning Algorithm Description

Navigation in dynamic environments is currently a problem because planners using reinforcement-learning techniques require assumptions about the behavior of dynamic agents. In this sense, the CADRL algorithm [47] proposes a navigation system focused on obstacle avoidance, without assuming any type of rule for the behavior of the agents. Moreover, it has no limitation in the number of agents for the observations. It is worth mentioning that the algorithm employed in our application was open source [48] and had been adapted to suit the requirements of our robotic application.

#### Algorithm Architecture

The reason why CADRL allows a variable number of agents is the inclusion of an approach used in natural processing language known as LSTM. Thus, a number of observations of the agents near the robot can be added. Meanwhile, observations concerning the robot are also counted. Finally, the network produces an output corresponding to the state value and to the policy.

Within the observations are included the position, velocity, radius and distance to the goal for the robot, while for the dynamic agents, the orientation, goal position and distance to another agent are also included. On the other hand, the action space is discrete, with 11 possible values. Also, the reward function is defined in Equation (10) [47]:

(10) $$R\_col=\left\{\begin{array}{cc} 1 & \mathrm{if}\;\mathrm{reached}\;\mathrm{the}\;\mathrm{goal}\\ -0.25 & \mathrm{if}\;d\_min<0\\ -0.1+0.05*d\_min & \mathrm{if}\;0<d\_min<0.2\\ 0 & \mathrm{otherwise}, \end{array}\right.$$

where d_min is the distance to the nearest agent.

### 3.5. Mapless-Deep-Reinforcement-Learning Algorithms Implementation

Traditional approaches to autonomous navigation commonly rely on accurate maps of the environment. However, in situations where a detailed map is not available, an algorithm that allows adaptability in such environments is critical. Given this problem, different DRL models were tested and compared in the Arena2D simulator [48].

#### 3.5.1. State Space

The state space consists of the following information:

- Distance and angle to the goal;
- 2D point cloud from the laser sensor;
- Distance and angle to the nearest obstacle.

It is worth noting that the 2D point cloud from the Lidar sensor was discretized into 240 samples, using the simulator’s tools. In this way, as the robot initially had a field of view of 240°, there was one sample for every approximately 1°. The choice of this value instead of a more partial degree sampling that would have less computational cost was due to the fact that the legs of the passers-by would be difficult to see for a lower-range scan per sample.

#### 3.5.2. Action Space

For the action space, linear velocities were taken in the range of [−0.4,0.4] m/s and angular velocities in the range of [−0.6,0.6] rad/s. Likewise, the algorithms were tested with both discrete and continuous action spaces, in order to analyze their influence on navigation performance. Table 4 presents the velocities for each possible robot action.

#### 3.5.3. Reward Model

First, the agent received a positive sparse reward equal to 100 if it reached the goal. Meanwhile, a dense reward was given as it got closer to or farther from that point, as seen in Equation (11) based on [16], where a k=10 was considered for the second reward:

(11) $$\text{Reward\_closeness}=\left\{\begin{array}{cc} 100 & \mathrm{if}\;\mathrm{reached}\;\mathrm{the}\;\mathrm{goal}\\ k\cdot (d_{t-1}-d_{t}) & \mathrm{otherwise}. \end{array}\right.$$

**Figure 11:**
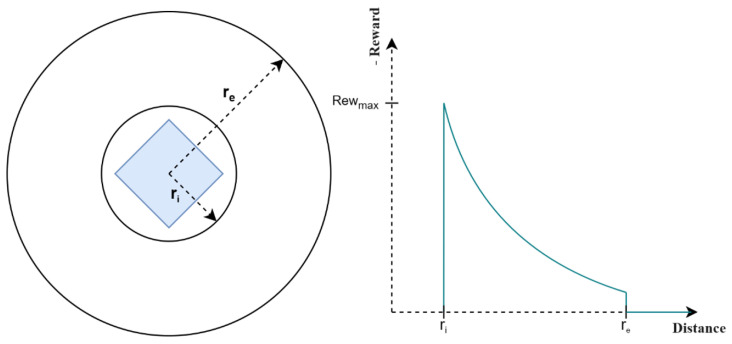
Characteristics of the reward model for obstacle avoidance.

The primary objective of the robot, in addition to reaching the goal, was obstacle avoidance in both static and dynamic environments. In this regard, a reward function was designed to assign a negative reward when the robot was at a certain distance from an obstacle, as depicted in Figure 11.

The exponential function allowed the reward to decrease significantly as the distance to the robot approached $r_{i}$. If the distance exceeded a certain value $r_{e}$, which corresponded to the robot’s safe zone, the reward became 0. Therefore, to achieve obstacle avoidance, the robot received a reward according to the set of Equation (12),

(12) $$\text{Reward\_avoidance}=\left\{\begin{array}{cc} -\text{Rew\_max}\cdot e^{-c\cdot (distance-r_{i})} & r_{e}\geq \mathrm{distance}\geq r_{i}\\ 0 & \mathrm{distance}>r_{e}: \end{array}\right.$$

- Where the parameter Rew_max=10 corresponded to the reward assigned to the robot when an obstacle was at a distance $r_{i}=0.335$.
- The parameter *c* allowed for controlling the fall of the exponential function. In this case, it was set to 5.
- The parameter $r_{e}$ was the most relevant for training, as it determined the start of the safe zone in which the robot no longer received a reward for the obstacle’s location.

To choose the parameter $r_{e}$, tests were conducted in an environment with static obstacles, taking into account that the total reward was the sum of the avoidance and closeness rewards:

(13) Reward=Reward_avoidance+Reward_closeness.

As shown in Table 5, as the value of $r_{e}$ decreased, the mean collision and average collision increased. This was because the robot would tend to navigate closer to obstacles, increasing the probability of collision. Therefore, the robot would have a lower ability to evade obstacles and would reach the goal instead of being stuck in an attempt to avoid an obstacle. The optimal value according to the table was 0.8, because the mean collision converged to the mean with $r_{e}=0.1$, and the success rate was higher than that of the latter. Additionally, the average reward was higher for this case.

#### 3.5.4. DRL Algorithm

In alignment with Figure 4, widely recognized algorithms such as DQN, PPO, and SAC were selected for each category of model-free models (value-based, policy-based, actor–critic).

- Deep Q Network (DQN) [49]: This algorithm is based on a value function—that is, the assignment of a numerical value for each state. A deep neural network is in charge of approximating this value, so that then the policy determines the action to be taken by means of the Equation (14), where the policy π chooses the action that maximizes the value function Q(s,a):
  (14) $$\pi \left(s\right)=\arg\max_{a}Q\left(s,a\right).$$
  DQN is an off-policy algorithm, because during training it employs a policy that is different from the optimal policy it is trying to estimate, because, first of all, in order to solve the problem of divergence generated by non-stationary targets, two instances of the neural-network weights are created. In this way, we have a network focused on saving an objective for multiple iterations and we have accumulated-experience data that allow us to treat the optimization problem as supervised learning.
- Soft Actor–Critic (SAC) [50]: This is an algorithm that belongs to the actor–critic category, whereby it is based on the estimation of a policy and a value function. Like DQN, SAC is also an off-policy algorithm, because it employs experiences from a behavior-focused policy that is different from the one used for optimization. SAC is well known for its ability to handle continuous action spaces, which is critical in environments where actions are not limited to discrete choices. In SAC, a deep neural network is used to model the Q function, which assigns a numerical value to each state-action.
  However, unlike DQN, SAC seeks to learn stochastic policies through the inclusion of an entropy term in the loss function. What this means is that the policy is not a deterministic function that maps states directly to actions, but rather produces probability distributions over actions. This component allows for randomness in actions and, thus, greater exploration in the environment. Consequently, it is important to emphasize that the agent’s objective is not limited to maximizing only the total expected reward, but also entropy. Thus, diversity in the agent’s behavior is promoted, in parallel to the optimization of the objective function.
- Proximal Policy Optimization (PPO) [51]: An algorithm that focuses on improving policies in sequential decision-making environments. Unlike the off-policy approaches discussed above, PPO belongs to the on-policy category, which means that it learns directly from the policy it is executing in the current environment.
  PPO, instead of approximating a value function, as in DQN, focuses on direct policy optimization. The goal is to find a policy that maximizes the cumulative reward over time, taking into account efficient exploration and exploitation. PPO addresses the policy-optimization problem in a more stable manner by limiting the policy updates at each iteration. This is achieved by imposing a constraint on the magnitude of policy change, which avoids drastic updates that could lead to learning instability.

### 3.6. Training Evaluation

**Figure 12:**
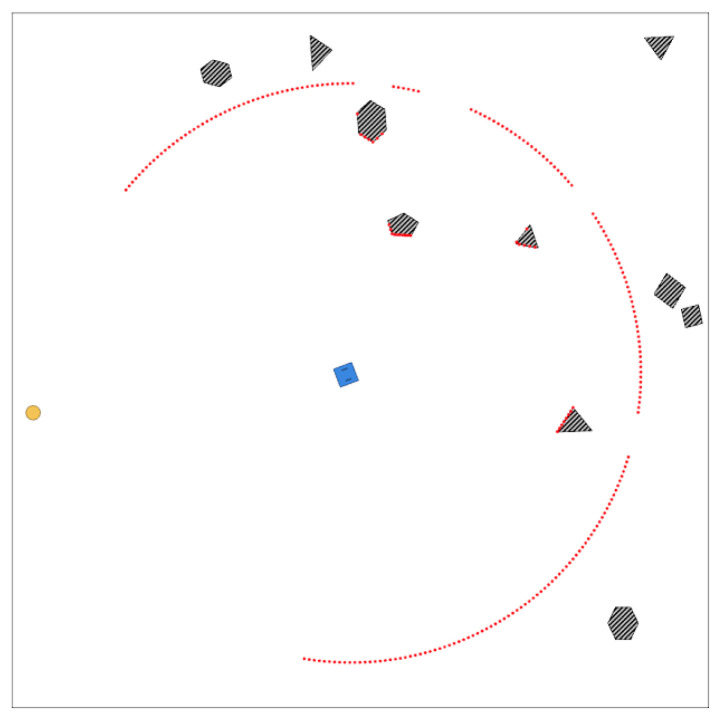
Training environment with stationary obstacles.

In order to obtain a model with a good performance in the autonomous-navigation task in the Arena2D simulator [48], different DRL algorithms were tested. For this purpose, as a starting point, 10 static obstacles were randomly placed in a quadrangular environment of dimension 24 m, as shown in Figure 12.

Additionally, in order to conduct a thorough comparison to traditional methods, we fine-tuned the hyperparameters for each algorithm. First of all, the learning rates were customized, with DQN being set at 0.001—a notably higher value compared to the 0.0003 assigned to PPO and SAC. This decision stemmed from the significantly longer training time needed for DQN compared to the other algorithms, as can be seen in Table 6. The higher learning rate was selected to accelerate the training process while maintaining a balance that avoided negatively impacting performance. Secondly, the discount factor (γ) had a notable impact on performance. It was observed that a value of 0.95 yielded optimal results for both DQN and PPO, while SAC exhibited superior performance, with a discount factor of 0.99. Finally, another parameter that held significance for DQN but not for the other algorithms was the exploration rate. To govern the exploration–exploitation trade-off, a value of 0.1 was assigned.

In Table 6, various evaluation metrics for the algorithms in the previously described environment are presented. It is worth noting that only for the DQN algorithm did the choice of a continuous over a discrete action space result in significant improvements. In general, DQN had a longer training time, which may have been due to the approximation it employed, based on approximating a Q-function, which can require a large number of iterations to converge to the optimal policy. Furthermore, SAC was the algorithm that showed the best combined results. In fact, SAC (off-policy) was more efficient in its samples than the PPO algorithm (on-policy). Consequently, it obtained a much lower collision rate, a lower training time, a higher average reward and had a slightly lower success rate than PPO. Finally, the SAC algorithm was trained using dynamic environments in the 2D simulator and then evaluated in Gazebo, as is detailed in the following chapter.

## 4. Results and Discussion

In the following chapter, a comprehensive comparative analysis was conducted, to juxtapose conventional autonomous-navigation algorithms with Reinforcement-Learning-(RL) methodologies. In this context, we selected DWA and TEB as the conventional local-planning methods of choice. This choice stemmed from the fact that DWA has been widely used as a benchmark in prior studies [52,53], serving as a reference point for evaluating and understanding the improvements and challenges offered by novel approaches. Conversely, TEB, also featured in previous comparison studies [52,53], has emerged as a significant enhancer of traditional-navigation algorithms, particularly in dynamic and non-stationary environments. Furthermore, TEB demonstrates superior computational efficiency compared to DWA [54], making it a suitable candidate for comparison to the most robust RL approaches available.

From the RL side, CADRL was chosen from among the other DRL approaches [55,56], because it proposes a navigation system focused on obstacle avoidance without assuming any rules in the behavior of the agents. Furthermore, SAC was chosen after testing and performing better among different DRL algorithms, as can be seen in Table 6. SAC merited inclusion in the comparison for its remarkable ability to function as a mapless algorithm while achieving comparable performance to map-based alternatives across several critical metrics.

### 4.1. Review and Definition of the Evaluation Environment

**Figure 13:**
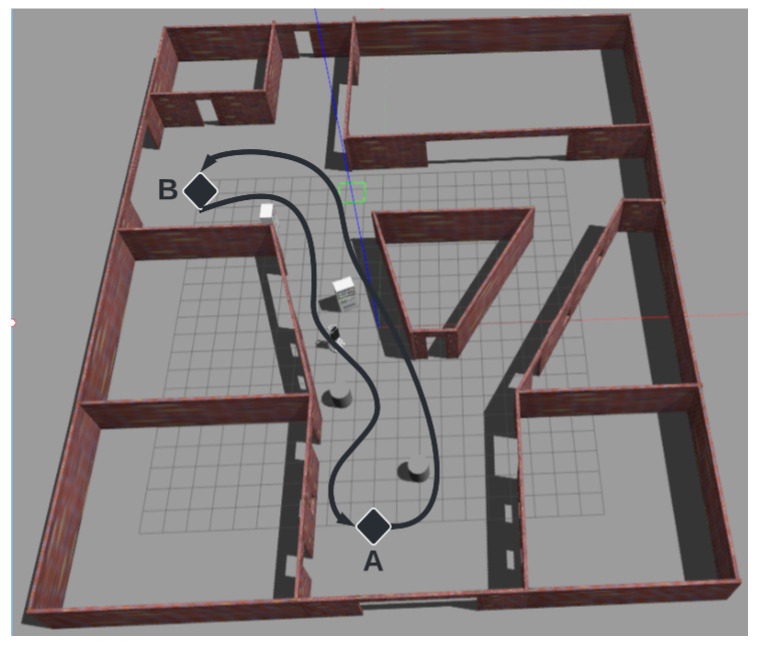
Test environment.

In order to evaluate the navigation algorithms, an environment and performance evaluation methodology was designed. To avoid the randomness of the results arising from taking into account different departure and arrival points for each iteration, routes between points A and B in both directions were considered, as shown in Figure 13. Ten attempts for each combination of parameters in the simulation were performed.

On the other hand, it should be noted that three types of environments were designed: empty, with static obstacles and with dynamic obstacles. For the empty environment, the world consisted only of passages and walls. In the environment with static obstacles, the number of obstacles was constant and equal to four. Finally, for the environment with dynamic obstacles, different parameters were varied, such as obstacle speed, type of movement, number of obstacles, etc.

**Figure 14:**
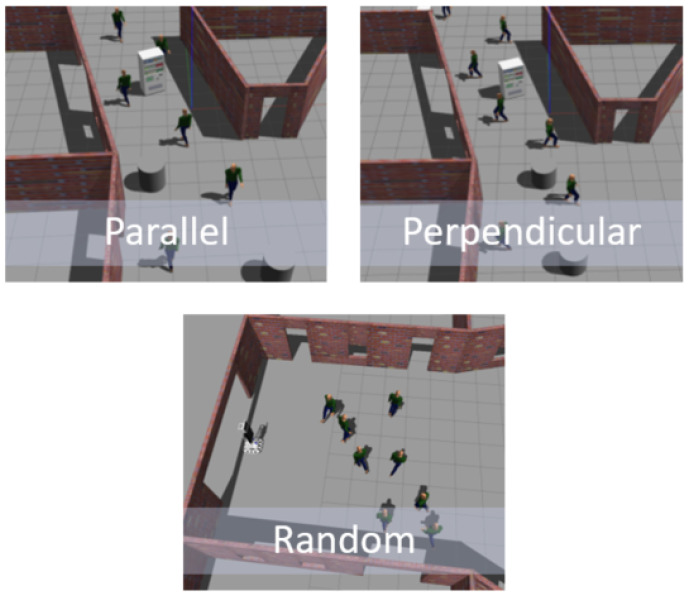
Movement types of dynamic obstacles.

The dynamic environment consisted of actors emulating the movement of passers-by, based on the social movements used in [57]. In that sense, the actors had three types of possible movements: parallel to the robot’s movement, perpendicular to the robot’s movement and random, as illustrated in Figure 14, where eight actors, each with a specified behavior, are shown in the simulated environment.

**Figure 15:**
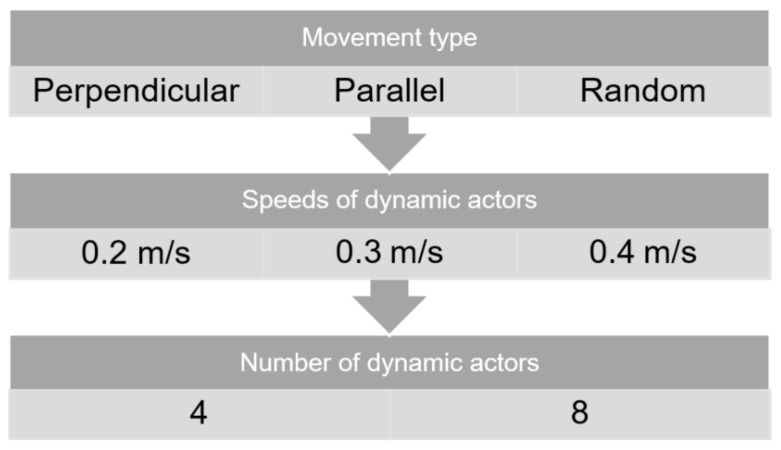
Variable parameters of the dynamic actors.

In each iteration, three parameters were varied fundamentally: type of motion, velocity and number of dynamic actors. As shown in Figure 15, the velocities of the actors were considered to be lower than the linear velocity of the robot, from 0.2 to 0.4 m/s. Likewise, the number of dynamic actors was chosen considering the dimensions of the designed world, in which four or eight passers-by were placed. It should be noted that the 10 evaluation iterations were for each of the possible combinations of the parameters of the three categories, so that a total of 180 samples were taken for each algorithm to be evaluated.

### 4.2. Definition of Evaluation Metrics

The evaluation metrics for the comparison of the different autonomous-navigation algorithms took into account trajectory and obstacle-avoidance efficiency. These metrics are shown below, with their respective units:

- Number of collisions;
- Safety score (%);
- Navigation time (s);
- Route length (m).

It is worth mentioning that, for navigation time, a route was considered unsuccessful if the robot became stuck in a recovery behavior for more than 15 s. Also, the metric used to evaluate the correct obstacle avoidance was the Safety score [54]. For this purpose, the total time spent by the robot on the route was divided into a given number of steps, the score was calculated by dividing the sum of the periods of time in which no pedestrian came within a distance $2\times r_{\mathrm{robot}}$ to the center of the robot, divided by the total number of steps, as shown in Equation (15):

(15) $${\mathrm{Safety}}_{\mathrm{score}}=\frac{\sum_{i=1}^{k}T_{i}}{T},$$

where $r_{\mathrm{robot}}$ represents an approximation of the robot radius, considering the platform base as circular, $T_{i}$ represents the ith time period during which the robot was not surrounded by any obstacle and *T* represents the total navigation time.

### 4.3. Performance with Non-Dynamic Obstacles

**Figure 16:**
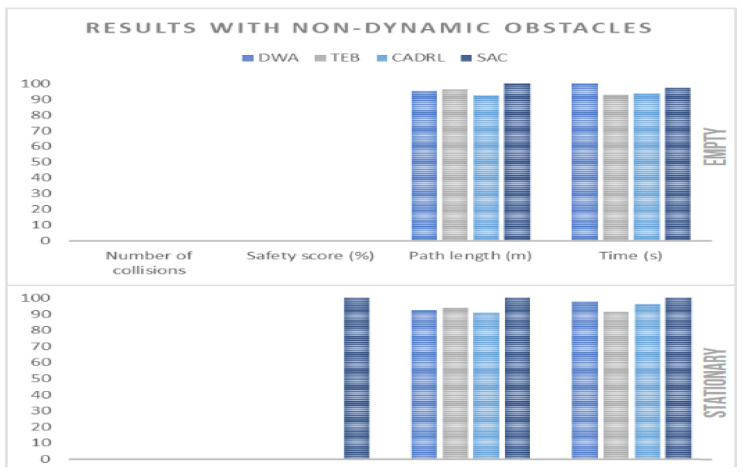
Plot for non-dynamic obstacles.

**Figure 17:**
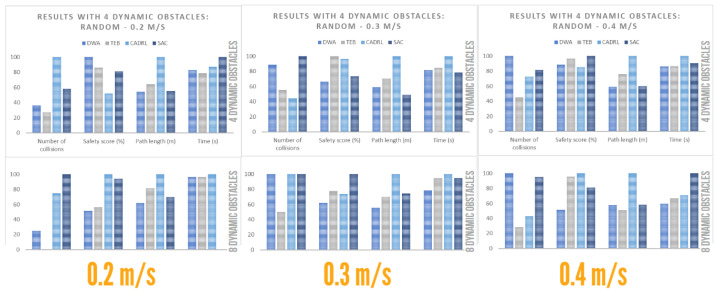
Plot for crossing social environment.

**Figure 18:**
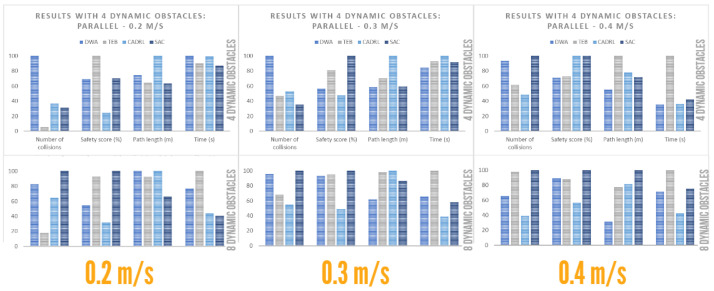
Plot for parallel social environment.

**Figure 19:**
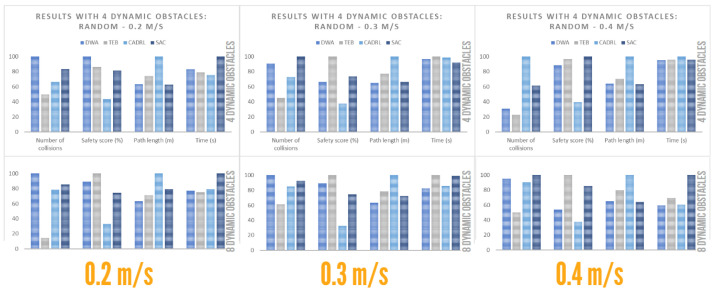
Plot for random social environment.

Next, the results for scenarios without dynamic actors were observed. For this purpose, the designed map was considered totally empty or with four stationary objects arranged along the robot’s path. It should be noted that 10 runs were performed for each type of algorithm and type of environment. It should also be noted that all the algorithms obtained 100% accuracy, i.e., they reached the goal in all the executions. From Table 7, graphs were generated to facilitate the comparison of the algorithms, as shown in Figure 16. It is important to highlight that in Figure 16, Figure 17, Figure 18 and Figure 19 the metric values have been normalized relative to the maximum value. This normalization allowed that a higher bar corresponded to superior performance in the respective metric. From this plot, the following conclusions were drawn:

- TEB provided a more optimal route on average, in terms of the navigation time.
- SAC had a less safe route on average, as it was the only algorithm that had a non-zero safety score for stationary environments.
- CADRL provided a more optimal route, in terms of distance traveled.
- All the algorithms provided collision-free routes.

### 4.4. Performance with Dynamic Obstacles

#### 4.4.1. Performance with Dynamic Obstacles: Crossing

The results for dynamic actors with a motion type crossing or perpendicular to the robot path are shown below. It should be noted that 10 runs were performed for each type of dynamic configuration and that the average of the metrics was calculated. Likewise, the TEB, CADRL and SAC algorithms obtained 100% accuracy, i.e., they managed to reach the goal. However, the DWA obtained an accuracy of 90% when the dynamic objects had velocities of 0.4 m/s, both for four and eight agents. From Table 8, graphs were generated to facilitate the comparison of the algorithms, as shown in Figure 17. The following conclusions were drawn, based on obstacle concentration:

- Low obstacle concentration:
  - DWA provided the second-shortest route with the highest temporal efficiency. However, its collision rate was higher for obstacles with a velocity of 0.3 m/s.
  - TEB provided the route with the least number of collisions, away from obstacles (high safety score). It also had high temporal efficiency.
  - CADRL, on average, provided the longest route with low temporal efficiency. Additionally, it exhibited a high collision rate.
  - SAC, on average, had the lowest safety score and the highest number of collisions but with high temporal efficiency.
- High obstacle concentration:
  - DWA provided high temporal efficiency and, on average, the shortest route but at the cost of a significant increase in the collision rate.
  - TEB provided the route with the least number of collisions, away from obstacles (high safety score). However, it significantly reduced its temporal efficiency compared to low obstacle concentration.
  - CADRL improved its collision rate and provided the second route with the lowest percentage. However, it offered the longest path and a low safety score.
  - SAC provided a route with high navigation time efficiency and a short route length. However, it had the highest collision rate and a fairly low safety score.

#### 4.4.2. Performance with Dynamic Obstacles: Parallel

The results for dynamic actors with a type of movement in parallel to or in the same direction of movement as the robot are shown below. It should be noted that 10 runs were performed for each type of dynamic configuration and that the average of the metrics was calculated. Likewise, the DWA and CADRL algorithms obtained 100% accuracy, i.e., they managed to reach the goal in all runs at the cost of an increase in the number of collisions. On the other hand, the TEB also obtained 100% accuracy with four dynamic agents; however, when this value increases to eight, it obtained an accuracy of 90% for speeds of 0.2 and 0.3 m/s and 80% for speeds of 0.4 m/s, while the SAC obtained an accuracy of 100%, except for speeds of 0.4 m/s, where it obtained 90%.

From Table 9, graphs were generated to facilitate the comparison of the algorithms, as shown in Figure 18. The following conclusions were drawn, based on obstacle concentration:

- Low obstacle concentration:
  - DWA provided, on average, the route with the highest number of collisions. However, it had high temporal efficiency, similar to the shortest navigation route.
  - TEB provided a route with the least number of collisions at low speeds. However, the number of collisions and navigation time increased, while the safety score decreased considerably at high speeds.
  - SAC had the highest number of collisions only at very high speeds; otherwise, it performed well. It also had the route with the best average safety score.
  - CADRL had the least number of collisions at high speeds but a low average for low speeds. However, its safety score was the lowest and it provided a long route.
- High obstacle concentration:
  - DWA provided the route with the shortest path length. However, its average collision rate increased significantly compared to a low-obstacle-concentration environment.
  - TEB provided a route with a low number of collisions, away from obstacles, except at high speeds. However, it reduced its temporal efficiency.
  - CADRL provided the route with the least number of collisions and better temporal efficiency. However, it had a low safety score.
  - SAC provided the route with the highest safety score. However, it had, on average, the route with the highest number of collisions.

#### 4.4.3. Performance with Dynamic Obstacles: Random

The results for dynamic actors with a random type of movement are shown below. It should be noted that 10 runs were performed for each type of dynamic configuration and that the average of the metrics was calculated. Also, even with random motion, all the algorithms except DWA in an environment of eight dynamic obstacles obtained 100% accuracy. This was mainly due to the fact that, despite being a more unpredictable motion for the robot, it presented a greater range of motion when moving within a region and not a linear motion.

From Table 10, graphs were generated to facilitate the comparison of the algorithms, as shown in Figure 19. The following conclusions were drawn, based on obstacle concentration:

- Low obstacle concentration:
  - DWA provided the second-shortest route.
  - TEB provided the route with the least number of collisions, away from obstacles. However, it had a long trajectory.
  - CADRL provided the longest route and, on average, the lowest safety score.
  - SAC provided a route with a high safety score. It also provided the shortest route.
- High obstacle concentration:
  - DWA provided the shortest route. However, the number of collisions increased significantly compared to an environment with low obstacle concentration.
  - TEB provided the route with the least number of collisions, away from obstacles.
  - CADRL provided an efficient route, in terms of navigation time. However, it had a low safety score and the longest path length.
  - SAC had the highest average collision rate and was the least efficient, in terms of navigation time.

## 5. Conclusions

This article presented a comparison study of indoor-autonomous-navigation algorithms for dynamic scenarios applied to mobile robots. Based on a detailed review, we selected two traditional-navigation algorithms (DWA and TEB) and two DRL-based algorithms (CADRL and SAC). We used a virtual robotic platform and a virtual environment to evaluate their performance and identify their advantages and disadvantages. Based on the results and performance of each algorithm, we provide the following conclusions:

- DWA provides a route with a high percentage of collisions, mainly due to its inability to generate reverse motion. However, it has, on average, good temporal efficiency and path length.
- TEB provides, on average, the route with the least amount of collisions and away from obstacles at low speeds and concentrations. However, at high speeds or concentrations, evasion and, consequently, temporal efficiency worsens.
- CADRL provides good obstacle avoidance in concentrated and fast environments, but at the cost of low temporal and routing efficiency.
- SAC provides a route with a high percentage of collisions, mainly due to the fact that it is a map-less algorithm. However, it has, on average, good temporal efficiency and path length.

Moreover, it is important to take into consideration the characteristics of the environment in which the robot must navigate. When navigating through dynamic environments the behavior of the actor can be different. For this reason, some conclusions can be described, based on the following results:

- For the exposed robot configuration, a dynamic environment where the movement of the actors is parallel to or in the same direction as the movement of the robot is more complicated to navigate. This is due to the limited range of vision, due to the dimensions of the robot, mainly in reverse movements.
- The most relevant dynamic parameter for social environments is the number of dynamic actors, as its variation entails a considerable reduction in the performance of the navigation algorithms.
- On average, metrics such as the number of obstacles, navigation time, path length and safety score increase as the number of dynamic agents and their speed increases.

As a result of this study, all the evaluated algorithms allowed the robot to navigate autonomously through a dynamic scenario. Moreover, the following recommendations can be stated for the usage of algorithms for autonomous navigation of ground-mobile robots in dynamic scenarios:

- When the information of the map is not available for the robot, it must be used with a mapless-DRL-based algorithm, such as SAC.
- If the map information is available and it is required to reduce the number of collisions even if the movement is slower, then a traditional algorithm, such as TEB, can be used.
- If the map information is available and it is required that the movement must be fast, then a traditional algorithm, such as DWA, can be used.
- If the map information is available and it is required that the trajectory must be the shortest, then a DRL-based algorithm, such as CADRL, can be used.

Based on the previous conclusions, we present some cases of practical applications for indoor dynamic environments, in order to determine the suggested algorithm. For any of the cases, if the information from the map is not available, the best option is to use a mapless-DRL-based algorithm:

- CASE 1: Robot for delivery of products in indoor environments (such as hospitals or offices): This type of application usually prioritizes the time it takes to deliver the products, for which it is recommended to use a traditional algorithm, such as DWA, or a DRL-based, such as CADRL, that uses shorter trajectories.
- CASE 2: Robot for marketing and interaction with users (in malls, universities, events, etc.): For any type of HRI applications, such as this one, it is recommended to use an algorithm that prioritizes the number of collisions, such as TEB, which is a traditional algorithm.
- CASE 3: Robot for security and inspection (in malls, offices, industrial plants or warehouses): This type of application usually prioritizes the shorter trajectories, in order to increase the robot’s autonomy. Hence, a DRL-based algorithm, such as CADRL, could be the best alternative.

This study was carried out in a virtual environment, in order to consider the different characteristics of the dynamic actors and their possible behaviors, which would not have been possible to do in a real environment. Through analysis in the virtual environment, it has been possible to evaluate multiple cases that could occur in a real environment. This will allow these results to demonstrate the functionality of the algorithms through tests in real environments, such as shopping centers, hospitals, offices or industrial plants.

As part of the future work for this research, the authors will implement the evaluated algorithms, using the robot in real use during the simulations and will evaluate its performance in navigating a mall and an office. Moreover, the evaluation will be performed by modifying the characteristic of each scenario, in order to evaluate the effect of generalization, especially with the DRL-based algorithms.

## Figures and Tables

**Table 1:** Comparison of the State-of-the-Art of local planners.

Algorithm	Publication	Scenario		Upgrade	Strongness	Tests
		Static	Dynamic			
DWA	Y. Lin et al. [32]	X	X	Fusion of Best-First Search (BFS) and DWA.	Improved performance in dynamic environments.	Simulation
	L. Tianyu et al. [33]	X		Modification of evaluation function for angle variation.	Improved reaction to obstacles and smoother trajectory.	Simulation, Real
TEB	M. Li and C. Yang [34]	X	X	Parameter setting and optimization method for the Ackerman model.	Ackerman obstacle avoidance.	Simulation
	Q. Dai and X. Ma [35]	X	X	Fusion A* and TEB for collision detection.	Reduced navigation time, route efficiency and performance in dynamic environments.	Simulation
MPC	S. Ishihara and M. Kanai [36]	X		Adaptability for multiple robots and unmapped environments.	No prior information on the environment is required.	Simulation
	J. Li and M. Ran [37]	X	X	Improved speed and trajectory planning.	Route efficiency and performance in dynamic environments.	Simulation

**Table 2:** Comparison of DRL-based-navigation algorithms (S: Static, D: Dynamic, SC: Social).

Publication	Navigation	Algorithm	Scenario			Perception Type			Actions Space	Test
			S	D	SC	RGB Camera	Depth Camera	2D Laser		
G. Chen et al. [8]	Map-Based	DQN	X	X		X	X	X	Discrete (28)	Simulation, Real
L. Liu, et al. [13]	Map-Based	A3C	X	X	X	X		X	Discrete (28)	Simulation, Real
Z. Lu and R. Huang [42]	Mapless	LDDPG	X	X	X			X	Continuous	Simulation
F. Leiva and Javier R. [43]	Mapless	DDPG	X	X				X	Continuous	Simulation, Real
R. Min-Fan and H. Sharfiden [44]	Mapless	DDQN	X	X		X	X	X	Discrete (5)	Simulation, Real
Y. Sasaki, et al. [45]	Map-Based	A3C	X	X	X			X	Continuous	Simulation, Real

**Table 3:** Common parameters for local planners.

Name	Notation	Value
Speed and acceleration limits	max_vel_x	0.4
	max_vel_theta	0.6
	acc_lim_x	2
	acc_lim_theta	0.1
Goal tolerance	xy_goal_tolerance	0.1
	yaw_tolerance	0.2
DWA parameters	sim_time	2.5
	vx_samples	20
	vth_samples	40
	path_distance_bias	32
	goal_distance_bias	24
	occdist_scale	0.02
TEB parameters	max_vel_x_backwards	0.4
	footprint_model_radius	0.335
	min_obstacle_dist	0.4
	include_dynamic_obstacles	True

**Table 4:** Discrete and Continuous Motion Commands.

Discrete	Continuous
Forward: (0.4m/s,0rad/s) Reverse: (−0.4m/s,0rad/s) Left: (0m/s,0.6rad/s) Left-Center: (0.3m/s,0.3rad/s) Right: (0m/s,−0.6rad/s) Right-Center: (0.3m/s,−0.3rad/s) Stop: (0m/s,0rad/s)	Linear velocity: [−0.4,0.4] m/s Angular velocity: [−0.6,0.6] rad/s

**Table 5:** Evaluation metrics for different values of $r_{e}$.

$r_{e}$	Mean Reward	Success Value (%)	Mean Collision
0.6	197.14	98.10	0.085
0.8	200.58	97.50	0.063
1	196.47	96.40	0.062

**Table 6:** Evaluation results for stationary environments.

Algorithm	Action Space	Mean Reward	Success Rate (%)	Mean Collision	Training Time
SAC	Continuous	200.58	97.50	0.063	1 h 4 min
PPO	Discrete	170.58	97.60	0.372	1 h 23 min
	Continuous	151.43	98.00	0.483	1 h 24 min
DQN	Discrete	170.29	84.00	1.360	1 d 15 h 58 min
	Continuous	161.79	80.90	0.089	1 d 11 h 47 min

**Table 7:** Results with non-dynamic obstacles.

	Scenario	Empty Map	Static Obstacles
Number of collisions	DWA	**0.0**	**0.0**
	TEB		
	CADRL		
	SAC		
Safety score (%)	DWA	**0.0**	**0.0**
	TEB		
	CADRL		
	SAC		1.57
Path length (m)	DWA	22.90	24.44
	TEB	23.24	24.85
	CADRL	**22.27**	**24.05**
	SAC	24.03	26.50
Navigation time (s)	DWA	64.70	69.51
	TEB	**60.07**	**64.95**
	CADRL	60.58	68.47
	SAC	63.05	71.08

**Table 8:** Dynamic Scenario 1—Perpendicular movement.

	Collisions Number	Safety Score (%)	Navigation Time (s)	Path Length (m)	Collisions Number	Safety Score (%)	Navigation Time (s)	Path Length (m)
$v_{\mathrm{obs}}=0.2\;m/s$	4 Dynamic Obstacles				8 Dynamic Obstacles			
DWA	0.7	7.41	80.78	27.10	2.8	18.01	**85.90**	**27.09**
TEB	**0.3**	**5.87**	**77.70**	29.13	**0.3**	**5.87**	91.00	32.27
CADRL	0.9	25.30	90.16	36.85	1.2	24.03	90.40	42.90
SAC	1.4	23.44	78.41	**26.89**	1.5	25.44	90.14	28.82
$v_{\mathrm{obs}}=0.3\;m/s$	4 Dynamic Obstacles				8 Dynamic Obstacles			
DWA	2.1	9.30	79.72	26.34	2.8	21.61	83.02	**25.90**
TEB	**0.5**	**7.33**	81.11	30.08	**1.4**	**15.33**	106.24	37.39
CADRL	1.4	22.60	87.27	42.98	1.8	29.65	90.25	43.05
SAC	2.0	22.97	**73.13**	**26.06**	3.9	35.00	**79.11**	27.30
$v_{\mathrm{obs}}=0.4\;m/s$	4 Dynamic Obstacles				8 Dynamic Obstacles			
DWA	1.3	9.45	77.06	26.34	2.5	23.97	89.43	27.98
TEB	**0.9**	**6.94**	82.93	30.48	2.2	**14.59**	107.20	36.93
CADRL	2.0	24.71	90.69	43.02	**1.9**	25.08	92.20	43.25
SAC	1.7	13.68	**73.11**	**26.04**	3.5	33.13	**76.80**	**26.80**

**Table 9:** Dynamic Scenario 2—Parallel movement.

	Collisions Number	Safety Score (%)	Navigation Time (s)	Path Length (m)	Collisions Number	Safety Score (%)	Navigation Time (s)	Path Length (m)
$v_{\mathrm{obs}}=0.2\;m/s$	4 Dynamic Obstacles				8 Dynamic Obstacles			
DWA	1.9	8.9	91.5	32.16	1.4	17.14	156.04	**26.50**
TEB	**0.1**	**6.15**	82.64	27.79	**0.3**	10.13	203.09	39.80
CADRL	0.7	25.00	90.70	43.17	1.1	29.67	89.19	43.13
SAC	0.6	8.77	**79.55**	**27.34**	1.7	**9.39**	**82.31**	28.33
$v_{\mathrm{obs}}=0.3\;m/s$	4 Dynamic Obstacles				8 Dynamic Obstacles			
DWA	1.7	18.25	**74.36**	**25.38**	2.1	15.78	142.50	**26.73**
TEB	0.8	12.65	82.21	30.34	1.5	15.47	215.74	42.41
CADRL	0.9	21.71	88.26	43.06	**1.2**	30.11	**83.25**	43.10
SAC	**0.6**	**10.31**	81.43	25.70	2.2	**14.70**	125.33	37.30
$v_{\mathrm{obs}}=0.4\;m/s$	4 Dynamic Obstacles				8 Dynamic Obstacles			
DWA	2.9	22.35	**83.51**	**25.97**	3.0	19.38	146.38	**26.36**
TEB	1.9	21.8	234.26	47.03	4.5	19.71	205.31	40.78
CADRL	**1.5**	15.81	85.29	36.74	**1.8**	30.64	**86.71**	43.03
SAC	3.1	**10.4**	98.24	33.84	4.6	**17.34**	154.99	52.65

**Table 10:** Dynamic Scenario 3—Random movement.

	Collisions Number	Safety Score (%)	Navigation Time (s)	Path Length (m)	Collisions Number	Safety Score (%)	Navigation Time (s)	Path Length (m)
$v_{\mathrm{obs}}=0.2\;m/s$	4 Dynamic Obstacles				8 Dynamic Obstacles			
DWA	0.6	**6.85**	89.16	26.97	1.4	10.97	86.82	**27.14**
TEB	**0.3**	7.95	84.88	31.63	**0.2**	**9.78**	**84.46**	30.73
CADRL	0.4	15.66	**80.96**	42.74	1.1	29.67	89.19	43.13
SAC	0.5	8.41	107.15	**26.86**	1.2	13.11	112.33	34.17
$v_{\mathrm{obs}}=0.3\;m/s$	4 Dynamic Obstacles				8 Dynamic Obstacles			
DWA	1	10.99	83.43	**26.62**	1.3	10.97	**80.19**	**27.16**
TEB	**0.5**	**7.29**	86.32	31.56	**0.8**	**9.78**	96.92	33.78
CADRL	0.8	19.38	85.22	40.84	1.1	30.10	83.24	43.10
SAC	1.1	9.92	**79.56**	27.23	1.2	13.11	96	31.06
$v_{\mathrm{obs}}=0.4\;m/s$	4 Dynamic Obstacles				8 Dynamic Obstacles			
DWA	0.4	9.62	**80.54**	27.52	1.9	21.34	**85.62**	27.86
TEB	**0.3**	**8.84**	81.17	30.29	**1.0**	11.50	99.54	34.34
CADRL	1.3	21.60	84.68	42.90	1.8	30.64	86.71	43.03
SAC	0.8	8.53	81.04	**27.09**	2.0	13.49	143.53	**27.59**

## Data Availability

The data presented in this study are available on request from the corresponding author.
